# A method for measuring the forces acting on a tree trunk using strain gauges

**DOI:** 10.1371/journal.pone.0245631

**Published:** 2021-01-15

**Authors:** Ayana Miyashita, Satoru Suzuki

**Affiliations:** Center for Forest Damage and Risk Management, Forestry and Forest Products Research Institute (FFPRI), Tsukuba, Ibaraki, Japan; Instituto Nacional de Investigacion y Tecnologia Agraria y Alimentaria, SPAIN

## Abstract

The wind force acted on a tree constantly changes in magnitude, direction, and distribution. We developed a method to measure simultaneously the amount of force (F), centroid of the distributed force (C), and direction of force (D) on a tree trunk using four strain gauges. F and C were estimated from the difference in the bending moments at two different positions along the long axis of the stem. D was estimated using the difference in the sensor outputs at two different radial positions at the same height. In principle, the two strain gauges should be oriented precisely 90° apart; however, this is unrealistic on an actual tree trunk. To calculate D, we developed a new method to detect the radial position and modulus of elasticity of each strain gauge after attaching it. We conducted three types of experiment. First, we loaded a wood pole with weights arranged in 11 patterns to test the accuracies of F and C for a distributed load. Next, we applied tensile forces to the wood pole and an evergreen conifer sapling from eight directions to test the accuracy of D, F, and C. On average, estimation errors were < 2% for both the distributed load and circumferential tensile load. Our method can estimate F, C, and D precisely, even if the wood is uneven and the strain gauges are not aligned. This is a great advantage for field wind force measurements.

## Introduction

Trees in fields are constantly exposed to wind forces. It has been suggested that mechanical stress caused by wind can determine tree architecture; stress exerted on the outer fiber of trunks or branches can be a determinant of their length or height versus diameter [1–3]. Furthermore, if the stress exceeds the mechanical strength of the trunk or root resistance, it causes fatal damage to the tree, such as trunk breakage or uprooting.

The wind profile is distributed along the height of a tree and can change in a moment. This means that the amount of force (F), centroid of force (C), and direction of force (D) on a tree trunk are always changing. These fluctuations result in a change in the bending moment, or mechanical stress, along the tree trunk and at the root. Therefore, to understand mechanical interactions between wind force and a tree, information of the specific distribution pattern of wind force exerted on the individual tree is essential. Such information is fundamental to understanding how and why wind damage occurs in a forest. Indeed, the amount of force and centroid of force differ among trees at different distances from the forest edge in wind tunnel experiments [4] and model simulations [5]. However, a practical method is lacking to measure actual wind forces on individual trees *in situ*. One prominent method of estimating wind force on a tree uses the drag coefficient of the tree and the wind speed at a height on the trunk [6, 7]. The drag coefficient is a dimensionless parameter used to quantify the resistance of an object in a fluid environment. Simply, the total wind (drag) force, F, can be expressed as F = 1/2ρAC_d_v^2^, where C_d_ is the drag coefficient, ρ is the density of air, A is the frontal area of the tree, and v is the mean wind speed. However, the wind speed profiles used are virtual and averaged, such as the logarithmic model [7], which does not express temporal changes of the wind profile. Determination of the drag coefficient is difficult and impractical for use in a forest stand; the drag coefficient of a tree changes with wind speed, crown morphology, and tree species in wind tunnel experiments [8–12] and large variation within individuals are observed in natural winds [9].

To determine the temporal pattern of wind force, we used a method involving strain gauges. The strain values can be directly converted into various mechanical properties, such as bending moment, mechanical stress, and modulus of elasticity. Strain gauges have been used previously in field measurements to investigate such mechanical values exerted on tree trunks or roots [13–18]. There is also a method using strain gauges to evaluate wind impact on a tree, in which the moment or mechanical stress exerted on a tree trunk is determined [19–22]. However, using only the moment, the amount of force and centroid of force cannot be separated. Among previous studies, Suzuki and Hayashi [23] described a method to estimate the amount of force and center of force using more than two strain gauges attached at different positions on the long axis of the specimen. However, according to their report, the estimation accuracy of the centroid of force was low on a standing tree. In addition, the direction of force has been estimated using two strain gauges at right angles to each other on the surface of a specimen [16, 24]; however, these estimation accuracies have been never tested.

In this study, we evaluated the accuracy of a method to measure the amount, centroid, and direction of force acted on a specimen using strain gauges. Our goal was to attain high estimation accuracy with uneven materials, such as living trees, even when the strain gauges are not aligned. For this, we first tested our method with a distributed load by setting a wood pole as a horizontal cantilever and loading it with several weights. Second, we tested circumferential loadings using the wood pole and an evergreen conifer sapling, both of which were set as vertical cantilevers. In these experiments, we applied a tensile force from eight directions and established a method to precisely calculate the direction of force together with the amount of force and the center of force. Finally, we discuss the merit of our method and future directions.

## Materials and methods

### Theory

#### The amount of force (F) and centroid of force (C)

**Fig 1:**
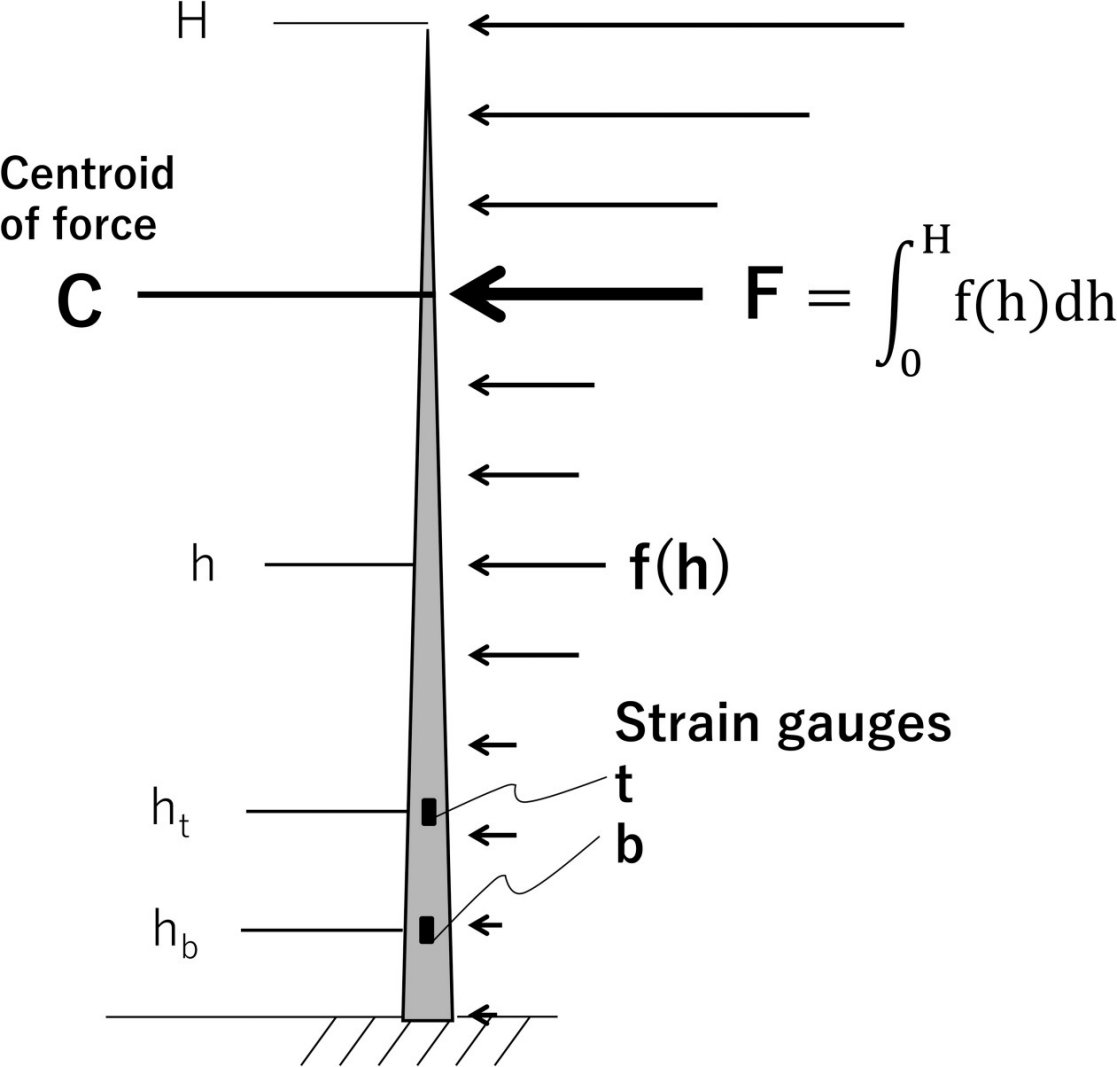
Schema for the wind force acting on a tree. Wind force has a distribution pattern. f(h), a force acting at height h; F, amount of distributed wind force; C, height of the centroid of the distributed force. Strain gauges are attached at h_t_ and h_b_. H is the tree height.

Part of a distributed load at height h of a tree, f(h), is schematically shown in Fig 1. The bending moments, M_t_ and M_b_ (units N m) at respective heights h_t_ and h_b_ (m; h_t_ > h_b_) on the trunk are determined as follows:

$$M_{t}=\int_{h_{t}}^{H}(h-h_{t})f(h)dh$$ (1)

and

$$M_{b}=\int_{h_{b}}^{H}(h-h_{b})f(h)dh$$ (2)

where H (m) is the tree height. The amount of distributed force, F (N), is obtained from the difference in the bending moments divided by the difference in the measured heights using Eqs (1) and (2) [23] as follows:

$$F=\int_{0}^{H}f(h)dh=\frac{M_{b}-M_{t}}{\left(h_{t}-h_{b}\right)}$$ (3)

where it is assumed that f(h) = 0 at height 0 ≤ h < h_t_; the assumption is reasonable when the moments are measured in the lower part of the trunk near the ground surface. M_t_ (Eq 1) is expressed as follows using the centroid of the force, C (m),

$$M_{t}=F(C-h_{t})$$ (4)

Therefore, C is shown in Eq (5) from Eqs (3) and (4).

$$C=\frac{M_{t}(h_{t}-h_{b})}{M_{b}-M_{t}}+h_{t}$$ (5)

The strain resulting from trunk deflection is proportional to the bending moment exerted on the trunk and is shown in Eq (6).

M=εEZ (6)

where ε is the strain (με), E is the modulus of elasticity of the sample (GPa), and Z is the section modulus of the cross-section (m^3^). Therefore, Eqs (3) and (5) can be written as follows:

$$F=\frac{\varepsilon_{b}E_{b}Z_{b}-\varepsilon_{t}E_{t}Z_{t}}{\left(h_{t}-h_{b}\right)}$$ (7)

$$C=\frac{\varepsilon_{t}E_{t}Z_{t}(h_{t}-h_{b})}{\varepsilon_{b}E_{b}Z_{b}-\varepsilon_{t}E_{t}Z_{t}}+h_{t}$$ (8)

Subscripts ‘t’ and ‘b’ indicate the heights h_t_ and h_b_, respectively. To calculate F and C, we measured ε_t_ and ε_b_ using strain gauges. E_t_ and E_b_ were determined beforehand in a pulling test by applying a known moment to the trunk while measuring the strain.

#### The direction of force (D)

A wind load acts on a tree from any direction in the field, while a strain gauge is fixed at a position on the trunk. Obtained strain values were proportional to the moments applied by the force components, which follows a trigonometric function of the direction of force. Then, a measured strain value, ε, is a function of the direction of the force, D (degrees), derived from Eq (6):

$$\varepsilon (D)=\frac{\left|M|cos(D-\theta_{g}\right)}{EZ}$$ (9)

where *θ_g_* indicates the angle of the fixed strain gauge from the origin set beforehand. D is derived by using the ratio of ε(D) values of the two strain gauges attached at different radial positions at the same height. The ratio is calculated as follows:

$$\frac{\varepsilon_{d2}(D)}{\varepsilon_{d1}(D)}=\frac{E_{d1}Z_{d1}cos(D-\theta_{gd2})}{E_{d2}Z_{d2}cos(D-\theta_{gd1})}$$ (10)

where the two strain gauges are indicated by subscripts d1 and d2. The cosine functions on the right side of Eq (10) are decomposed by the addition theorem. Then, the numerator and denominator on the right side are divided by cosD to obtain tanD (= sinD / cosD). Eq (10) is re-arranged as follows:

$$tanD=\frac{{\varepsilon_{d1}(D)E}_{d1}Z_{d1}cos\theta_{gd2}-\varepsilon_{d2}(D)E_{d2}Z_{d2}cos\theta_{gd1}}{{\varepsilon_{d2}(D)E}_{d2}Z_{d2}sin\theta_{gd1}-{\varepsilon_{d1}(D)E}_{d1}Z_{d1}sin\theta_{gd2}}$$ (11)

D is the arctangent of Eq (11) and is determined at h_t_ and h_b_; D_t_ is obtained from strain gauges td1 and td2, and D_b_ is obtained from strain gauges bd1 and bd2. The values of D_t_ and D_b_ were consistent in this study; for more precise estimation, we applied D_t_ to obtain M_t_ and D_b_ for M_b_ in Eq. (9).

**Fig 2:**
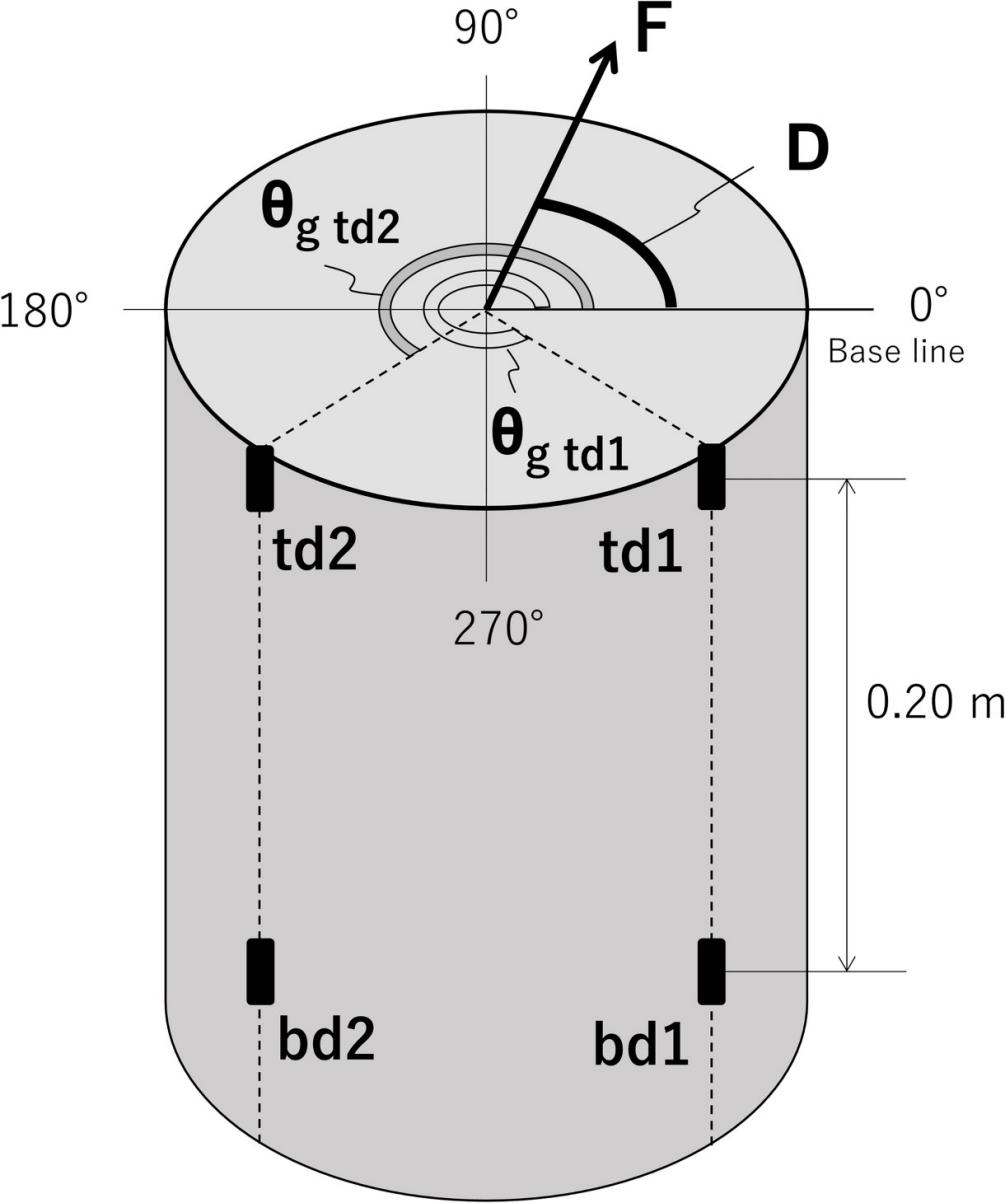
Schema of the arrangement of the strain gauges. A cross-section of a specimen at height h_t_ is shown. In the panel, D gives the radial direction of the force, F, from baseline. θ_g td1_ and θ_g td2_ are the radial positions of strain gauges td1 and td2, respectively. bd1 and bd2 are the strain gauges at height h_b_.

To measure F, C, and D simultaneously, two strain gauges at different heights on the trunk and two additional strain gauges in different radial directions on the trunk are required. Therefore, four strain gauges were arranged on the tree trunk (Fig 2). These strain gauges were named td1, td2, bd1, and bd2. The initial ‘t’ and ‘b’ indicate the heights h_t_ and h_b_, respectively; ‘d1’ and ‘d2’ indicate different radial positions at the same height.

#### Determining the position of the strain gauge and the E value

Accurate E values at the positions of the strain gauges are necessary to estimate F and C, and precise values of θ_g_ are also essential for estimating D. The following equation is obtained by transforming Eq (9):

$$\frac{\varepsilon (D)Z}{\left|M\right|}=\frac{1}{E}cos(D-\theta_{g})$$ (12)

**Fig 3:**
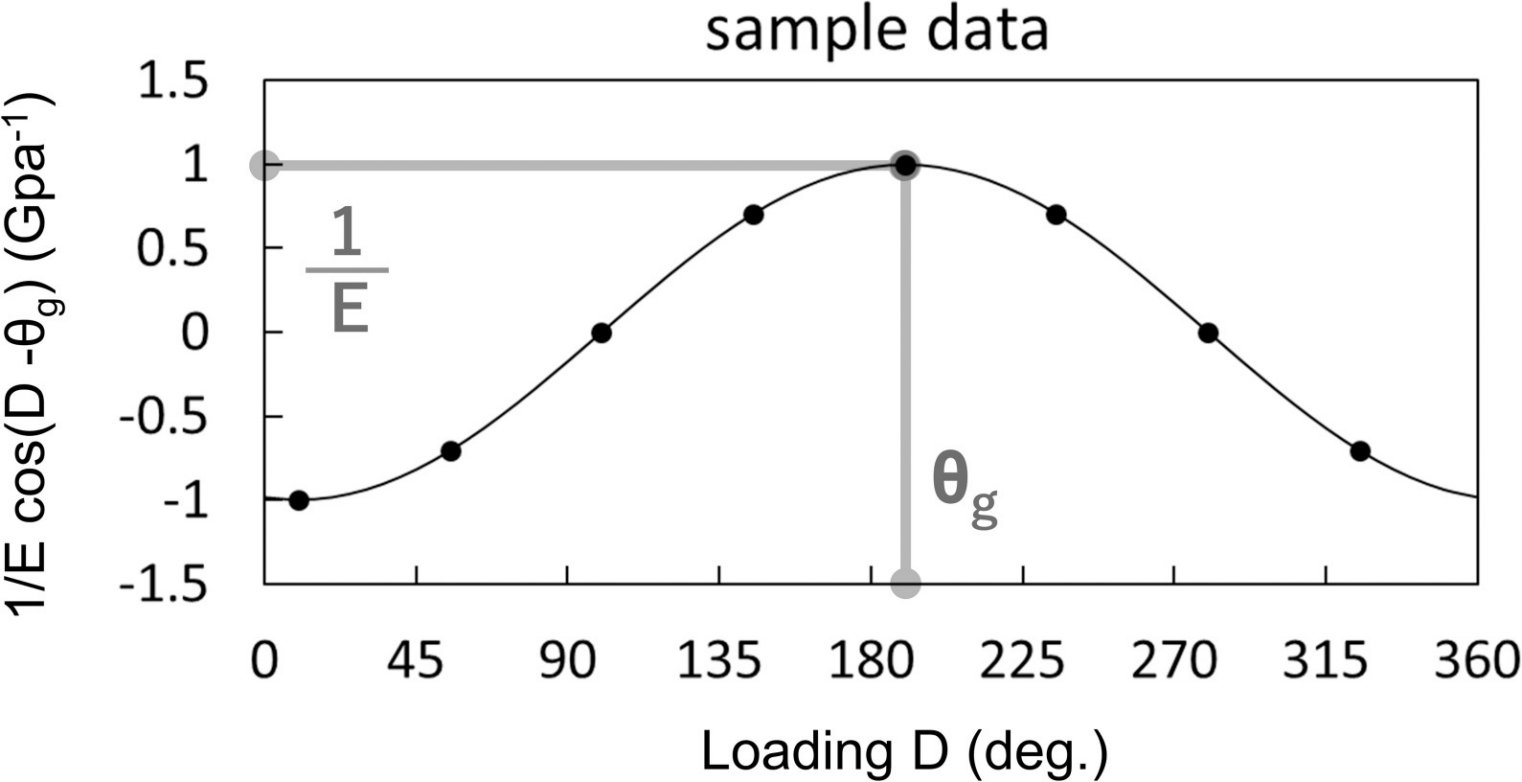
Schema of the fitted curve derived from the pulling test for eight directions. D shows the loading direction of the force from baseline. The phase shift of the curve gives θ_g_, and the amplitude of the curve gives 1/E.

From the curve formed by Eq (12), we obtain two parameters (Fig 3): the phase shift of the curve gives θ_g_, and the amplitude of the curve gives 1/E. Based on this, we performed a pulling test applying known moments to our specimens from eight known D (for details, see *Circumferential load experiments* in *Experimental design*). We plotted the eight obtained sets of ε(D)Z/|M| and D and regressed them to the cosine function 1/E*cos(D—θ_g_), using the least squares method. A curve was determined for every four strain gauges. Each test was iterated three times for the wood pole and sapling experiments described below.

To show the utility of our method for detecting θ_g_ and E, we compared the accuracy of the estimates with those determined by the following parameter assignments: ‘right angle’, the angle between strain gauges d1 and d2 was set as 90°, and E was estimated for every gauge using Eq. (12); ‘common E’, E values were averaged and used as a fixed value for all strain gauges, and θ_g_ was determined individually using Eq. (12); and ‘right angle & common E’, the angle was fixed at 90°, and E was fixed as an averaged value.

### Experimental design

#### Specimen and apparatus

An air-dried wood pole and the aboveground portion of a sapling were used for our experiment. The wood pole was 0.01 m in diameter and 0.9 m in length, made from *Chamaecyparis obtusa* (an evergreen conifer). The sapling was *Cryptomeria japonica* (an evergreen conifer), grown in the field, and was ~ 3 m in height with a straight and circular cross-section of the stem. For the experiment, we used the top 182 cm of the sapling.

Four strain gauges (FLA-3-11-5L, Tokyo Measuring Instruments Laboratory, Tokyo, Japan), named td1, td2, bd1, and bd2, were attached to the surface of each specimen using cyanoacrylate adhesive. The distance between strain gauges t and b was 0.2 m (Fig 2). The difference in the radial direction between strain gauges d1 and d2 was ~ 90°. A 1.5 × 1.5-cm section of the bark and cambium layer was removed from the sapling before gauge attachment. Several previous works used metal hinges with which strain gauges were attached to tree trunks [13, 15, 17, 25, 26]. We, however, attached strain gauges to the specimen without hinges because our measurement time of several weeks was relatively short-term; thus, we could eliminate the costs of the hinges. The strain gauges were connected to a data logger [EDS-400A (wood pole) or EDX-2000A (sapling), Kyowa Electronic Instruments, Tokyo, Japan] through a bridge box (DB-120T-8, Kyowa Electronic Instruments). The sampling time interval was 0.1 s, and instantaneous values were recorded.

#### Distributed load experiment

**Fig 4:**
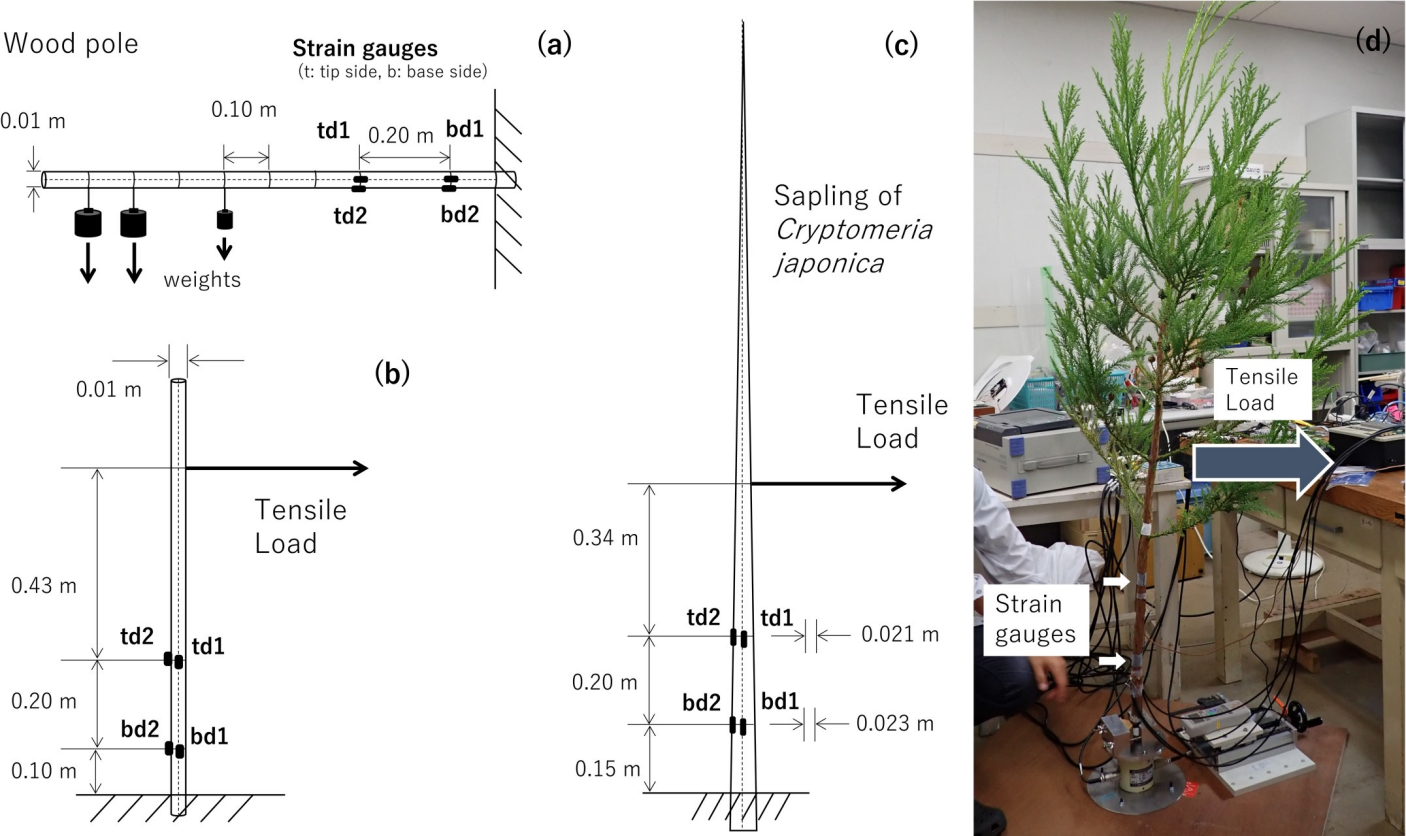
Schemata and photograph of the tests. (a) Distributed force experiment, (b) wood pole test, and (c) and (d) sapling test.

We applied several weights to the wood pole to mimic a distributed load. The wood pole was set horizontally as a cantilever to be easily loaded using several weights (Fig 4A). Five loading points were established at 0.1-m intervals along the long axis. The weights were 0.5–2.0N, and several combinations of one to five weights were installed at the loading points. In total, the number of cases for combinations of the loading F and C was 11 (Table 1). Each treatment was repeatedly measured at least six times.

**Table 1:** Distributed load experiment.

Test No.	Loading F (N) at each loading point*					Total loading F (N)	Loading C* (m)	Number of loading weights
	1	2	3	4	5			
**1**	0.98	-	-	-	-	0.98	0.095	1
**2**	-	-	0.98	-	-	0.98	0.294	1
**3**	-	-	-	-	0.98	0.98	0.493	1
**4**	0.98	-	0.98	-	-	1.96	0.195	2
**5**	0.98	-	-	-	0.98	1.96	0.294	2
**6**	0.98	-	0.98	-	0.49	2.45	0.254	3
**7**	0.49	-	0.98	-	0.98	2.45	0.334	3
**8**	0.98	-	0.49	-	0.98	2.45	0.294	3
**9**	0.98	0.98	0.49	0.20	0.20	2.85	0.211	5
**10**	0.98	0.20	0.49	0.20	0.98	2.85	0.294	5
**11**	0.98	0.49	0.98	0.20	0.20	2.85	0.229	5

* Distance from the t-strain gauges. The distances between the loading points and t-strain gauges were 0.095, 0.194, 0.294, 0.393, and 0.493 m. F, amount of force; C, centroid of force.

#### Circumferential load experiments

We applied a horizontal tensile load to the wood pole and the sapling. Each specimen was set vertically as a cantilever fixed at the bottom (‘wood pole experiment’, Fig 4B, and ‘sapling experiment’, Fig 4C and 4D). Each specimen was pulled by a firm string connected to a digital force gauge (DS2-5N, IMADA, Toyohashi, Japan). We applied loads of ~ 0.5, 1.0, 1.5, and 2.0 N. Each treatment was iterated three times. The treatments were conducted from eight radial directions (loading D) approximately every 45°. Each D was also recorded as an angle from baseline. The pulling tests were repeated on three different days.

From the outputs of the strain gauges, we determined E and θ_g_ for each strain gauge for each of the 3 days. To estimate F, C, and D during the pulling test from the strain values, we used the parameters for the same day.

## Results

### Accuracy of the distributed load

**Fig 5:**
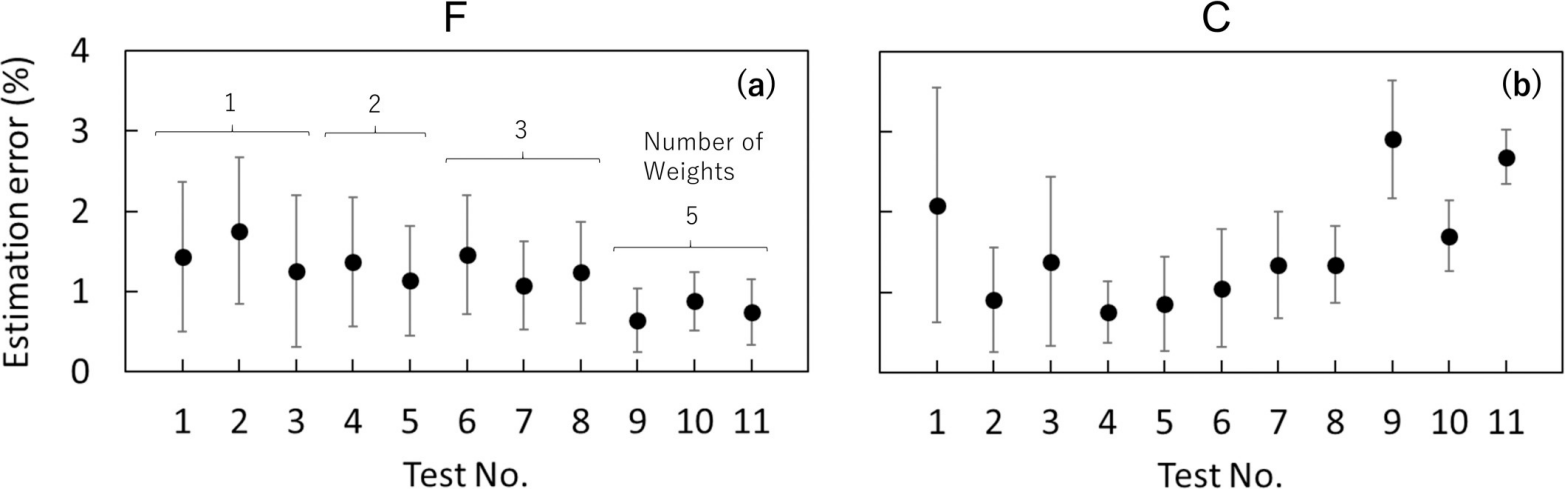
Estimation errors for the distributed load experiment. (a) The amount of force, F, and (b) the centroid of the distributed force, C, shown in the order of the distributed loading tests (for the details of each test, see Table 1). The number of loading weights is the same in panels (a) and (b). The estimation error for F and C was defined as the absolute value of [1 –(estimated value) / (known value)] × 100 (%).

**Fig 6:**
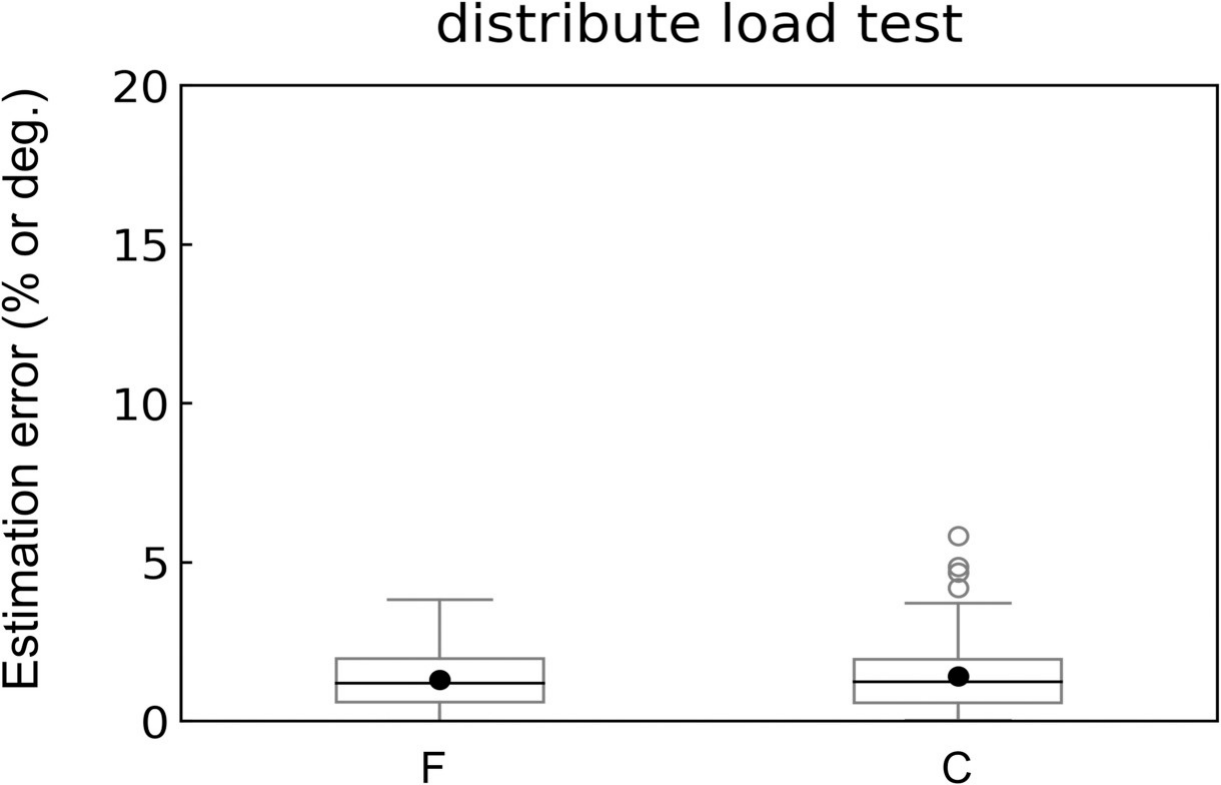
Box plots of estimation errors for the distributed loading experiment. F, the amount of force; C, the centroid of force. Each black solid circle in the panel shows the average value. For the calculation of the estimation errors, see the caption of Fig 5.

The errors in F tended to decrease as the number of loading weights increased; the average estimation errors of F were 0.6–0.9% when weights were distributed at all points (Fig 5A). The average estimation errors of C were relatively higher (1.7–2.9%) when the weights were distributed at all points (Fig 5B). Overall, the mean and median errors were approximately 2% (Fig 6), although outliers appeared in approximately 5% of the C errors.

### Positions of the strain gauges and E values

**Fig 7:**
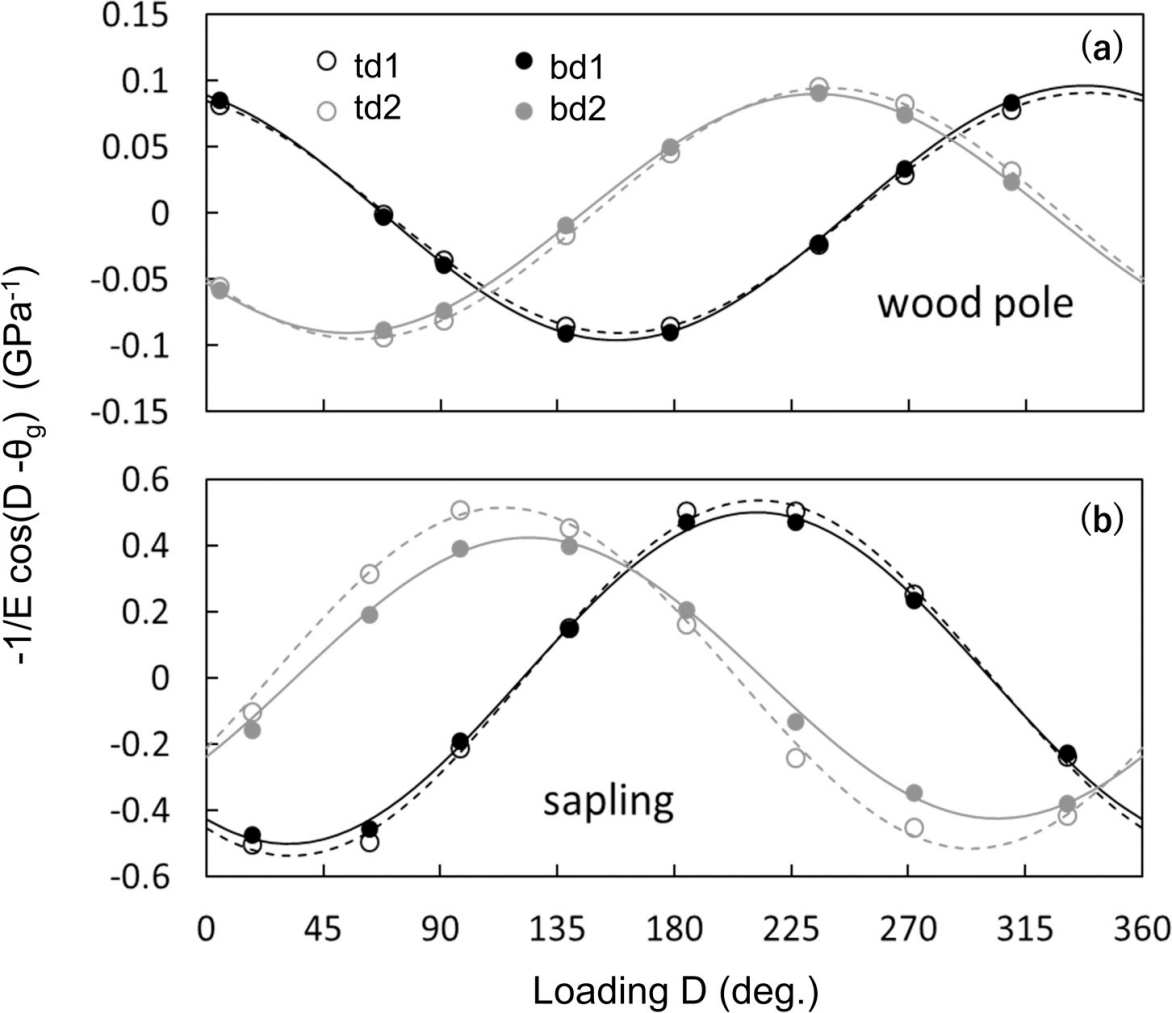
Curves of 1/E*cos(D-θ_g_). The results for the (a) wood pole and (b) sapling tests. Here, we used the function with negative sign, -1/E* cos(D-θg), because this form is adapted to outputs of a strain gauge which gives a positive value for tension and a negative value for compression. For each panel, circles show the measurement values from each rotation and lines show the fitted curves. D (degrees) is the radial direction of loading F, θ_g_ (degrees) is the direction of each strain gauge on the specimen, and E (GPa) is the modulus of elasticity. Black open circles and black dashed lines, strain gauge td1; black solid circles and black solid lines, strain gauge bd1; grey open circles and grey dashed lines, strain gauge td2; and grey solid circles and grey solid lines, strain gauge bd2. For the E and θ_g_ values of each strain gauge, see Table 2.

For both the wood pole and sapling experiments, values of ε(D)Z/|M| were regressed well (R^2^ ≥ 0.99) by the trigonometric function 1/E*cos(D—θ_g_), with a period of 2π (Fig 7). Among the strain gauges, there was a significant difference in E values within a specimen for both the wood pole and sapling (Table 2, Fig 7). For θ_g,_ there were significant differences of approximately 10° from a right angle between strain gauges d1 and d2 at h_t_ and h_b_: 99.9–103.0° for the wood pole and sapling, except for the angle between bd1 and bd2 of sapling, which was 89.2° (Table 2). The difference in radial direction was relatively small between td1 and bd1: 1.3° for the wood pole and 0.9° for the sapling. However, there were relatively greater differences between td2 and bd2: 4.2° for the wood pole and 9.8° for the sapling.

**Table 2:** E and θ_g_ for each strain gauge.

Strain gauge	Wood pole			Sapling		
	E (GPa)	θ_g_ (deg.)		E (GPa)	θ_g_ (deg.)	
**td1**	11.2 ± 0.15 ^a^	159.6 ± 0.82	^n.s.^	1.9 ± 0.01 ^a^	32.6 ± 0.21	**
**bd1**	10.6 ± 0.15 ^b^	158.3 ± 0.77		2.0 ± 0.01 ^b^	31.7 ± 0.17	
**td2**	10.5 ± 0.10 ^ab^	59.5 ± 0.59	**	1.9 ± 0.03 ^a^	292.7 ± 1.06	**
**bd2**	11.0 ± 0.08 ^ab^	55.3 ± 0.73		2.3 ± 0.02 ^c^	302.5 ± 1.07	

Numerical values are the average ± SD of three iterations of the test. Different letters indicate significant differences (*p* < 0.05, Tukey’s test).

** statistically significant (*p* < 0.01, *t*-test).

### Accuracy of the circumferential load

**Fig 8:**
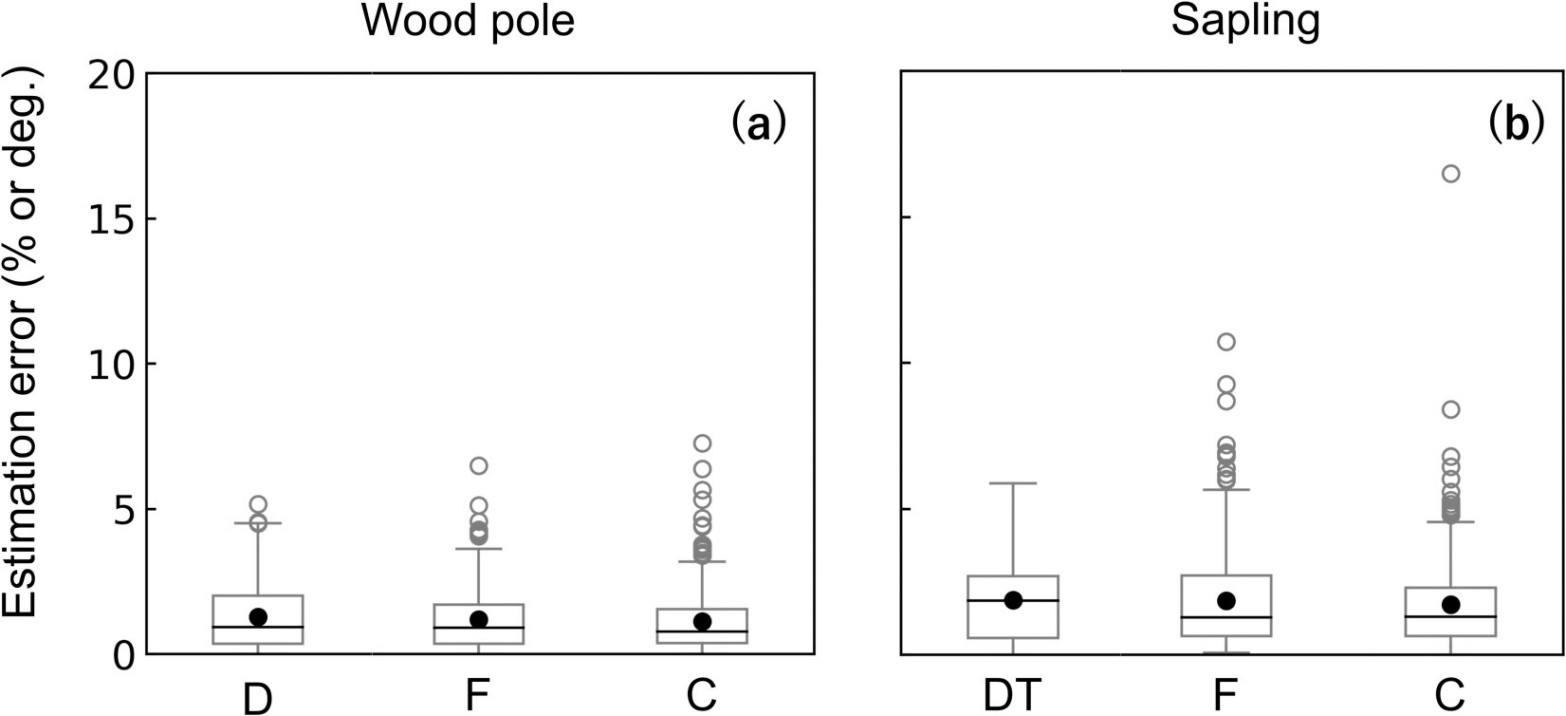
Box plots of all averaged estimation errors for the circumferential experiments. Results for the (a) wood pole and (b) sapling tests. F, the amount of force; C, the center of force; and D, the direction of force at h_t_. Each black solid circle in the panel shows the average value. For the calculation of the estimation errors for F and C, see the caption of Fig 5. The estimation error for D was defined as the absolute value of (known value)–(estimated value) (degrees).

**Fig 9:**
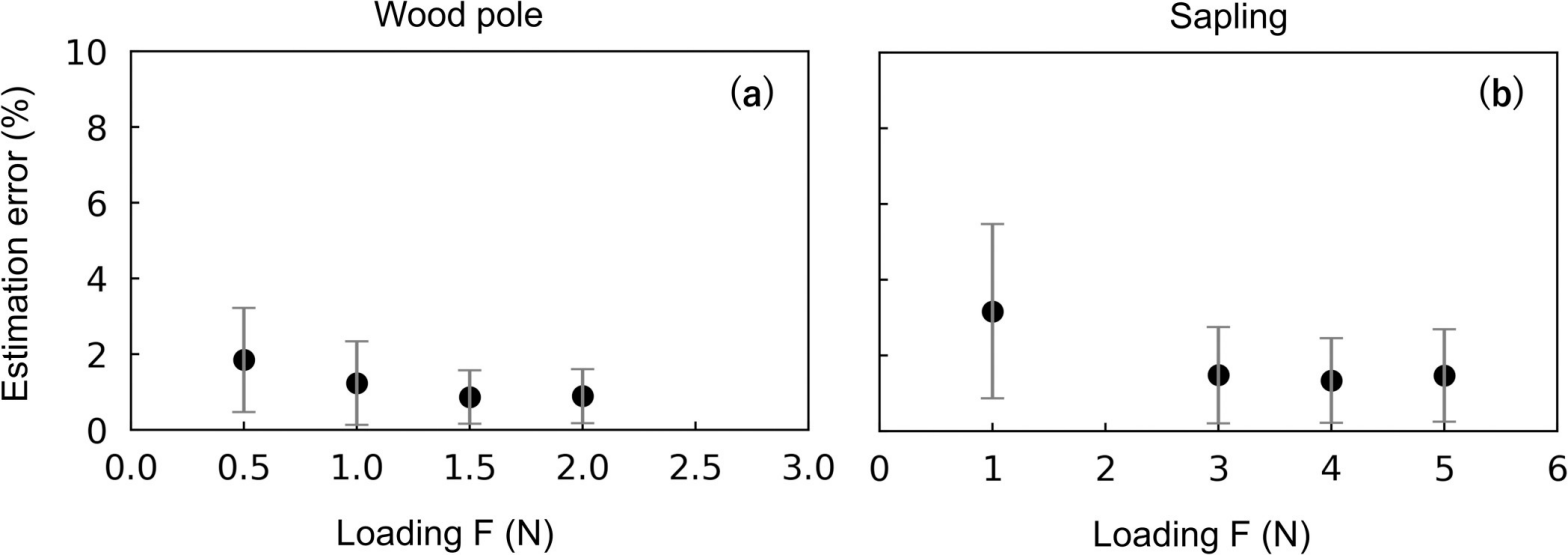
Estimation errors for the amount of force, F, versus the known loading values of force for the circumferential experiments. (a) Wood pole and (b) sapling. For the calculation of the estimation errors, see the caption of Figs 5 and 8.

**Fig 10:**
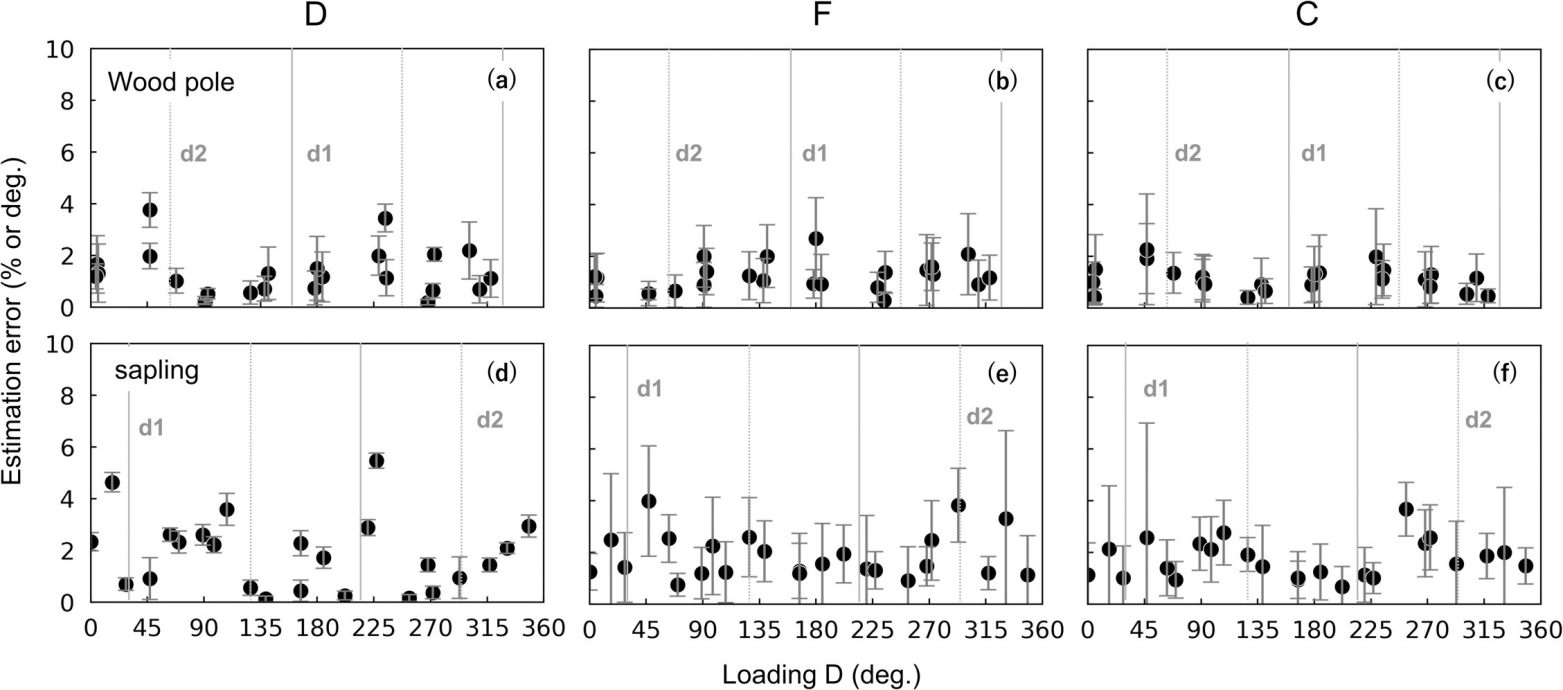
Estimation errors versus the known loading direction of force for the circumferential loading experiments. The estimation errors of (a, d) the direction of force at h_t_, D, (b, e) amount of force, F, and (c, f) the gravity center of force, C. Results for the wood pole (a–c) and sapling (d–f). Grey solid lines and ‘d1’ on the panel show the radial position and the radial position + 180° of d1 strain gauges; grey dotted lines and ‘d2’ show the radial position and the radial position + 180° of d2 strain gauges. For the calculation of the estimation errors, see the caption of Figs 5 and 8.

For both the wood pole and the sapling experiments, average estimation errors were < 2% or 2° (Fig 8). The sapling experiment showed slightly larger errors compared with other experiments. For both experiments, the estimation errors of F were significantly large when the loading F was smallest (Fig 9). However, the overall estimation errors were ≤ 2% and ≤ 4% throughout the loading F for the wood pole and sapling experiments, respectively. The loading D might have affected the estimation errors, especially in the sapling experiment (Fig 10D–10F), but the regularity was not clear. Fig 10 shows that the estimation errors for D were < 4° for the wood pole and < 6° for the sapling; for F and C, the estimation errors were < 3% for the wood pole and < 4% for the sapling.

### Effect of detecting θ_g_ and E on accuracy

**Fig 11:**
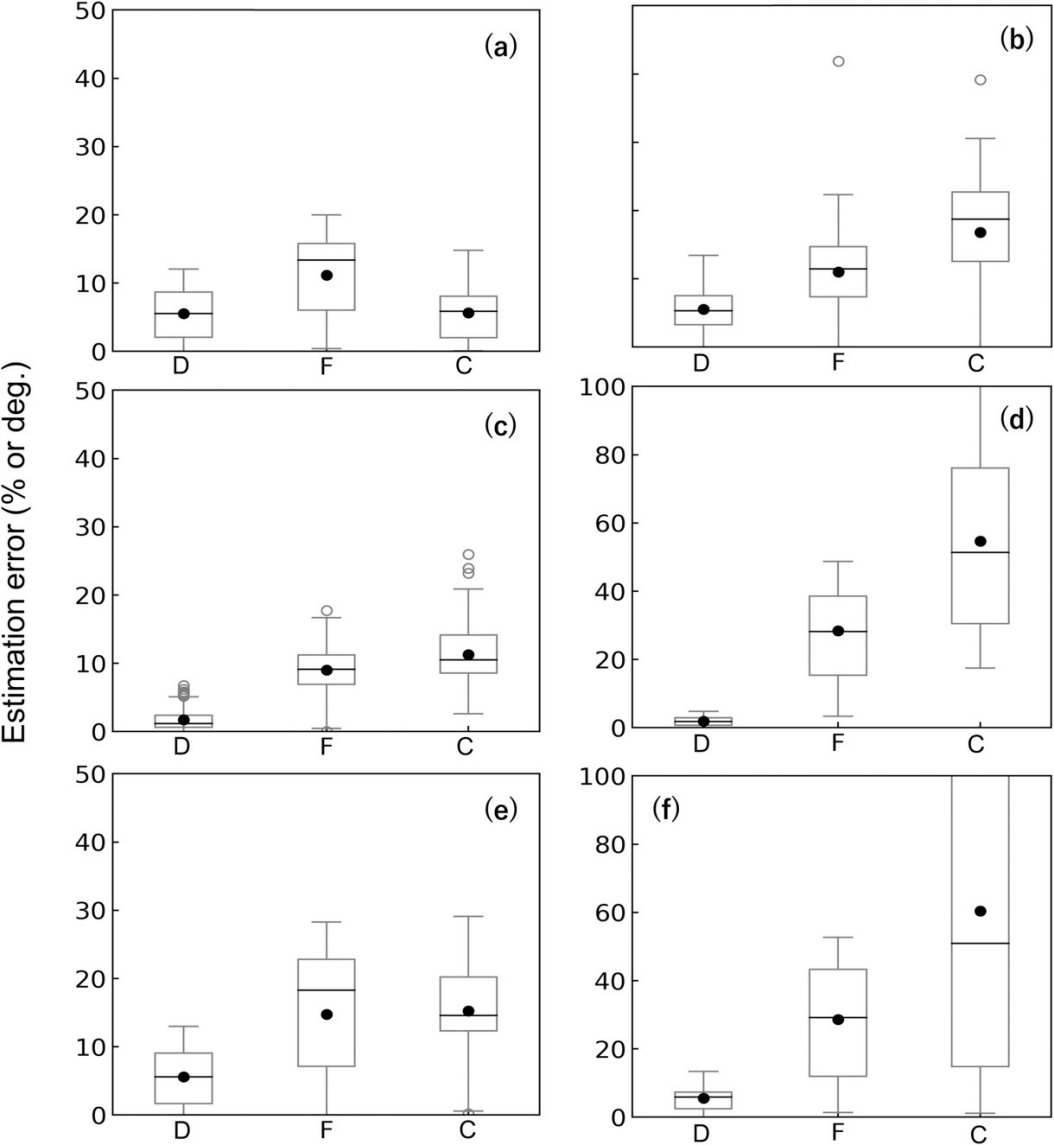
Box plots of all averaged estimation errors for circumferential experiments calculated using parameter assignments other than those used in our method. (a, b) The ‘right-angle’ parameter assignment in which the angle between strain gauges d1 and d2 was assumed to be 90° and the forces were estimated based on vector synthesis of outputs from the gauges. (c, d) The ‘common E’ assignment in which we used the average of the modulus of elasticity, E, from each strain gauge for the calculations of F, C, and D. (e, f) The ‘right angle & common E’ assignment is a combination of the first and second methods. Results for the wood pole (a, c, and e) and sapling (b, d, and f). F, the amount of force; C, the center of force; and D, the direction of force at h_t_. Each black solid circle in the panel shows the average value. For the calculation of the estimation errors, see the caption of Figs 5 and 8.

For the parameter assignment ‘right angle’, the estimation errors were larger than our estimation (Figs 8 & 11A and 11B). For example, the estimation errors of D, F, and C of the wood pole were approximately 9, 10, and 5 times larger than those with our method, respectively. For ‘common E’, the estimation errors of D were comparable with those of our method (Fig 11C and 11D). However, the respective errors of F and C were 8 and 10 times larger for the wood pole and 15 and 32 times larger for the sapling compared with our method. For the case ‘right angle & common E’, the errors in F and C were prominent. For the wood pole, the errors in F and C were 13 and 6 times larger, respectively, and for the sapling, they were 15 and 35 times larger than the errors determined with our method. For the estimation errors of C of the sapling, the averaged values and variance were very large for all parameter assignments.

## Discussion

### Estimation of the distributed load

The fact that we could precisely estimate F and C regardless of the pattern of the distributed load indicates that the method can be used for field measurements, where the wind force working on a tree can have any distribution pattern along the trunk.

From the results, the errors in F were smallest when the loaded weights were distributed at all five points. This result was considered to be due to the magnitude of the load rather than the load distribution because smaller errors were observed for greater amounts of F in both the wood pole and sapling experiments (Fig 9) and the amount of the loading weight was greater in cases when more loading points were used (Table 1). For estimation errors of C, a similar trend was observed. We could not determine the reason because there was no relationship between the estimation errors and the amount of loading F or loading C.

### Position of the strain gauges and E values

Our results indicate that there is technical difficulty in attaching several strain gauges precisely on the surface of a circular column. In this study, the angle between strain gauges d1 and d2 was not precisely 90° in most cases. For the strain gauges t and d at the same direction, the radial positions were relatively consistent, but were not exactly the same. However, positioning accuracy of the strain gauges is not necessary for our method, because the position is detected and used as is.

For E values, the observed variance among the strain gauges on the same specimen strongly suggests that the materials themselves were not uneven. It is known that within a trunk there are uneven patterns of specific gravity, reaction wood, and fiber orientation; these are associated with mechanical properties of the wood [27]. The discordance was larger for the sapling (Table 1, Fig 6). From these results we note that a method dealing with some irregularity in a material is essential to measure wind force on a living tree.

In this study, an apparent value of the modulus of elasticity, E, was obtained. For the wood pole, observed E values were comparable with the reported value [28]. For the sapling, as a living trunk of *C*. *japonica*, values of E were approximately 1/3 to 1/2 of the reported values in green timber [29, 30]. Note that in our experiment E can be underestimated by ~7% because of the self-weight which can increase actual bending moment on the trunk. Nevertheless, the E values were relatively small. However, there are almost no reports for E of an entire trunk of small diameter. In our preliminary observation, E was estimated to be 1.8–2.4 GPa for seedlings and saplings of the species (unpublished data). Such small values of E may be a general feature of saplings of the species.

### Estimation of force from any direction

Our method was demonstrated to be able to estimate force acting from any direction. Overall, the estimation errors were small, and there were no clear patterns affected by the loading D. Observed estimation errors were larger for the sapling experiment than the wood pole experiments (Figs 8–10). This may be due to the unevenness of the material, as suggested by the variance in E values. However, the errors were still sufficiently small.

The high estimation accuracy is dependent on a significant feature of our method in which E and θ_g_ are determined for each strain gauge and used for the estimation. This was proved paradoxically by the results of the three simplified parameter assignments: ‘right angle’, ‘common E’, and ‘right angle & common E’. The results of the ‘right angle’ assignment demonstrate that detecting E for each sensor contributes to the precise estimation of F and C, especially for the sapling (Figs 8 & 11A and 11B). The results of the ‘common E’ assignment clearly demonstrate that detecting the radial positions of the sensors greatly improves the estimation accuracy of D (Fig 11C and 11D). However, just by identifying θ_g_, the estimation errors of F and C remained higher. As shown in the result of the ‘right angle & common E’ assignment (Fig 11E and 11F), if we did not determine both θ_g_ and E for each strain gauge, the estimation errors were largest. For the sapling, the estimation errors and variances of F and C were quite large using the ‘common E’ and ‘right angle & common E’ methods. The fact that our method greatly improved the estimation accuracy for the sapling indicates that it is advantageous for measurements of actual trees in the field.

### Application to field measurements and future directions

Future directions include, first, testing our method under natural winds with temporal fluctuations of velocity and direction, and second, adapting the method more for real trees. Regarding the second issue, our method is not adapted currently to a huge force; therefore, it is not suitable for large leaning trunks. In this study, the maximum observed strain values were ~ 1000 με. In this relatively small range, the mechanical stresses were proportional to the strain values, and the tree’s own weight would have a negligible effect on the relationship. However, when a larger force acts on it, the linearity will be lost, and the weight of the tree would alter the stress–strain relationship. It will also be necessary to adapt our method to trunks with non-circular cross-sections, which is often observed in mature trees. In such cases, we may need an additional function to those used in the current method.

Wind damage is a problem in both natural and managed forests in many regions of the world [31]. To predict risks, it is fundamental to evaluate the balance between the mechanical strength of a tree and wind force. Although there are many studies providing datasets for the resistance of a tree to uprooting or trunk breakage (e.g., [32–35]), there are few datasets for real wind forces acted on individual trees. In mechanical models developed to predict the wind damage risk of a stand, such as HWIND [36] and ForestGALES [6], the wind force applied is simulated based on averaged wind speeds around the site. In those models, however, the prediction accuracy for individual trees is not satisfyingly high, at approximately 50–60% [37]. In the current model, to improve the damage prediction, many field factors are considered in the calculation, such as gusts of wind, individual tree measurements, and distances among trees (e.g., [22]). However, there are no actual data of wind force exerted on a tree. Collecting such data will contribute to furthering our understanding of the mechanisms of wind damage and improve the prediction accuracy of risks.

## Conclusion

We proposed a method to measure the amount of force, F, centroid of force, C, and direction of force, D, acting on a tree simultaneously using strain gauges attached to the trunk surface. In this study, we showed that it is difficult to attach strain gauges at precise positions and to align each sensor on actual wood or living trees, and that these can lead to large estimation errors. In our method, the radial position, θ_g,_ and modulus of elasticity, E, were detected for each strain gauge after attaching them to the trunk surface. Using our method, we showed that precise estimation is possible for the distributed load and tensile load from eight directions. First, in the pulling test, the position and E of each strain gauge are described as a trigonometric function of the loading D. Then, from two strain gauges attached to different radial positions at the same height, an accurate D is calculated. Using the calculated D, the precise value of the bending moment can be determined. Then, F and C, which are calculated from the difference in the moments at different heights, are also determined with high accuracy.

## Supporting information

S1 Data Strain data and other relevant data of the distributed load experiment. (XLSX)

S2 Data Strain data and other relevant data of the wood pole in the circumferential load experiment. (XLSX)

S3 Data Strain data and other relevant data of the sapling in the circumferential load experiment. (XLSX)

S4 Data Modulus of Elasticity (E) and radial position (θg) determined for each strain gauge on each experimental day. (XLSX)

S5 Data Estimation errors calculated by our method and by threes simplified parameter assignments for the wood pole in the circumferential load experiments. (XLSX)

S6 Data Estimation errors calculated by our method and by threes simplified parameter assignments for the sapling in the circumferential load experiments. (XLSX)
